# Effect of Dialectical Behavior Therapy on Negative Affect, and Symptoms of Depression and Anxiety in Individuals with Borderline Personality Disorder during COVID-19 Pandemic

**DOI:** 10.3390/jcm13092603

**Published:** 2024-04-29

**Authors:** Olga Malas, Alba Gómez-Domenech

**Affiliations:** 1 Department of Psychology, Sociology and Social Work, University of Lleida, 25001 Lleida, Spain; 2 Terres de Lleida Mental Health Centre, 25008 Lleida, Spain

**Keywords:** borderline personality disorder, dialectical behavioral therapy, negative affect

## Abstract

**Background**: This study investigated the effectiveness of dialectical behavior therapy (DBT) in patients with borderline personality disorder (BPD) during the COVID-19 pandemic, assessing negative affect, depression, and anxiety levels as indicators of health. **Methods**: A total of 287 participants were recruited, including BPD patients at different stages of treatment and the general population without a diagnosis of BPD. Questionnaires were used to assess the fear of COVID-19 and the referenced health indicators. **Results**: No differences were observed between groups in levels of fear of COVID-19, but there were differences in the health indicators studied. BPD patients in long-term treatment showed levels of negative affect similar to those of the general population, while those in early treatment stages exhibited significantly higher levels. However, no significant improvements were observed in levels of depression and anxiety in the long-term treatment group compared to those who underwent the initial treatment phase. **Conclusions**: These findings underscore the importance of effectively intervening in BPD, especially in stress-inducing situations such as the pandemic, and suggest the need to explore complementary approaches to addressing depression and anxiety in this clinical context.

## 1. Introduction

Borderline personality disorder (BPD) has an estimated worldwide prevalence of 0.7 to 5.8% in the general population [1], 3% in adolescents, and 1.5% in the elderly [2,3], affecting 10% of ambulatory psychiatric patients and 15% to 20% of psychiatric inpatients [4,5]. In general, this condition represents a significant challenge, for the patient, their family, and the medical system [6], due to the severity of its symptoms, the frequent presence of concurrent conditions, and the high treatment dropout rate [7,8].

BPD presents a wide range of symptoms, including micropsychotic episodes, emotional instability, impulsivity, dysfunctional self-image, feelings of worthlessness, fear of abandonment, self-harm, and suicide attempts [9,10]. Self-destructive behaviors have been reported in around 70% of those affected, with suicide rates approximately 50 times higher than in the general population [3,11]. At the same time, there are references suggesting possible differences between genders [12]. As example, men appear to be more prone to endorsing criteria of intense and inappropriate anger and impulsivity, and higher levels of substance abuse, while women tend to endorse criteria of chronic feelings of emptiness, affective instability, and suicidal/self-harming behaviors, and higher levels of depression, anxiety, and obsessive–compulsive symptoms [13,14,15,16,17]. 

One of the most extensively studied evidence-based treatments for BPD patients is dialectical behavior therapy (DBT) [18,19,20], which has shown significant decreases in hopelessness, depression, anger expression, and suicidal ideation [21,22]. DBT has also demonstrated effectiveness in stabilizing and controlling self-injury and high-risk behaviors [23,24], as well as in reducing the number of hospitalizations and suicide attempts [25]. Initially developed as a treatment for highly suicidal women, it was later refined for the treatment of BPD [26]. DBT draws upon principles from behavioral and cognitive-behavioral psychotherapy, dialectical philosophy, and Zen practices [26]. The systematic reviews conducted in 2006, 2010, 2021, and 2024 [26,27,28,29] support the effectiveness of DBT for patients with BPD regarding self-injurious behaviors, depression, and suicidality. Both individual and group DBT promote the development of psychosocial and motivational skills that help foster adaptive and functional behaviors [30]. 

DBT conceptualizes BPD as a comprehensive disorder rooted in the emotion regulation system, stemming from a combination of biological predisposition to emotional vulnerability and emotional learning within an invalidating environment [26]. Thus, the observed efficacy of DBT could be mediated by improvements in behavioral control and emotional regulation [31,32], which would be enhanced by skills training [30,33,34]. The therapy aims to assist patients in finding a balance between acceptance and change, with the overarching goal of enabling them not only to survive but also to build a life worth living [26]. However, studies investigating the impact of DBT on specific emotions are notably limited, and primarily focus on anger and fear/anxiety [35]. Several studies suggest that DBT yields significant reductions in both state and trait anxiety [36,37,38]. Additionally, research indicates that individuals with BPD and comorbid post-traumatic stress disorder exhibit decreases in feelings of shame, guilt, and fear following DBT intervention [37,39], although these emotional changes may be specific to this particular comorbidity [35].

From a biosocial theory perspective, behavioral and cognitive dysfunction in BPD is intricately linked to emotions, with many of the behavior issues associated with the condition stemming from underlying emotional vulnerability and dysregulation [40]. Thus, compared to healthy participants, individuals with BPD report more frequent, intense, unstable, and persistent negative emotional responses [41,42,43], as well as greater emotional reactivity to negative interactions [44,45]. For instance, individuals with high levels of BPD symptoms demonstrate heightened impulsivity in a behavioral task compared to those with low BPD symptoms, but only following induction of negative emotions [46]. Similarly, individuals with elevated BPD symptoms reveal deficiencies in social problem-solving relative to healthy counterparts, but again, only after exposure to negative emotions [47]. These and other examples related to the subject lead to the conclusion that patients with BPD may be capable of acting quite skillfully at times, and only under conditions of intense emotion do they act impulsively or struggle to resolve interpersonal difficulties [40]. However, studies employing laboratory paradigms to examine how individuals with BPD respond to real-time emotional cues have yielded contradictory results, and it remains unclear under what conditions heightened emotional vulnerability can be expected in BPD [40].

Considering that people with BPD appear to be more sensitive to social stressors [40], the COVID-19 pandemic provides a very suitable real-life situation in which to study the effectiveness of an intervention in patients with BPD, which may help shed light on this topic. Therefore, this study was conducted during a critical phase of the COVID-19 pandemic in Spain, which resulted in prolonged confinement of the population. This confinement led to significant changes in daily routines and habits, which, combined with uncertainty and fear, negatively impacted the mental health of the population [48], with an increase in the prevalence of stress, anxiety, and depression [49,50,51]. Additionally, several studies reported that psychiatric patients or those with previous mental pathologies experienced significantly higher levels of anxiety, depression, and stress compared to the general population, which were associated with increased impulsivity and greater suicidal ideation [52,53,54]. Within psychiatric disorders, individuals with BPD are characterized by experiencing high emotional dysregulation and possessing inadequate coping strategies for managing stress and negative affect [55,56]. It has been demonstrated that individuals with BPD exhibit greater difficulties in emotional regulation and an increased tendency towards impulsive responses during situations of uncertainty [57]. Thus, the uncertainty, isolation, concerns, and economic risks associated with the pandemic represented a potentially hazardous context that could have a significant psychological impact on this population cohort [58,59]. 

The relationship between the inability to label emotions and psychological distress occurs in both BPD patients and healthy individuals, although it is more significant for the BPD group because these individuals experience negative emotions more frequently [60]. Furthermore, they have a lower tolerance for discomfort, which is understood as the ability of an individual to pursue a goal while experiencing negative emotions [61]. Interindividual differences in the effects of perceived risk toward COVID-19 infection (i.e., fear of COVID-19) on anxiety and distress intolerance predicted both anxiety and depressive symptoms [62] during the pandemic. Considering the behaviours commonly exhibited by those with low distress tolerance, it is reasonable to propose that such patients may be less likely to engage in distressing aspects of the treatment program and therefore receive fewer benefits, as evidenced by a worse response to treatments [63]. 

In this way, studies conducted during the first wave of COVID-19 demonstrate considerable mental distress among BPD patients [64,65]. Negative feelings of anxiety, sadness, and helplessness were the most frequent immediate reactions [64]. However, while social distancing could provide feelings of safety from the virus, reactions to social isolation, acceptance of restrictions, and adaptability varied, ranging from feelings of relief to heightened feelings of isolation and loneliness, as well as symptoms of depression and negative thoughts [65]. Therefore, levels of negative affect, anxiety, and depression can be considered a reflection of the distress caused by the pandemic in patients with BPD.

In Spain, home confinement was imposed from 15 March to 20 June 2020. During the confinement, mental health centers treated patients by telephone and electronically. From that date, in-person visits were resumed under strict preventive measures to avoid infections during the sessions (hygiene, masks, distancing, etc.). In January 2021, during the execution of this study, Spain was experiencing a second wave of the disease, with a significant increase in the number of cases, hospitalizations, and deaths, carrying the consequent risk of relapse of BPD symptoms due to an increase in pandemic-related distress.

Thus, the objective of the study was to determine whether there is greater emotional reactivity in BPD in a real context, coinciding with a critical phase of the COVID-19 pandemic, among patients undergoing DBT, compared to patients in first stage of treatment and the general population. Our hypothesis was that (1) patients diagnosed with BPD, undergoing fewer than two therapy sessions, are expected to show significantly higher levels of fear of COVID-19, negative affect, anxiety, and depression compared to both the healthy general population without a diagnosis of BPD and patients with BPD undergoing long-term DBT (with more than 20 therapy sessions). Furthermore, (2) the BPD cohort undergoing long-term DBT was anticipated to demonstrate levels of fear of COVID-19, negative affect, anxiety, and depression similar to those observed in the healthy general population group without a BPD diagnosis.

## 2. Materials and Methods

### 2.1. Participants

In this study, 287 adults participated, including 64 men (22.3%) and 223 women (77.7%), with a mean age of 40.08 years (*SD*: 12.07), ranging from 18 to 68 years old. The majority of the sample had a university education (77%) or professional training (20.7%). Of the total sample, 82.2% were employed, 7.4% had lost their job due to the COVID-19 pandemic, and 10.4% were not employed before or during the study. Regarding living arrangements, 12.6% lived alone. 

The sample was divided into three groups. The experimental group consisted of 69 patients, 5 men (21.7%) and 54 women (78.3%), with an average age of 37.33 years (*SD*: 12.32), diagnosed with BPD, and receiving DBT. The control group consisted of 30 patients, 6 men (20%) and 24 women (80%), with an average age of 39.63 years (*SD*: 12.16), diagnosed with BPD, but who had completed a maximum of two sessions. The third group was composed of 188 volunteers from the general population, 43 men (22.9%) and 145 women (77.1%), with an average age of 41.15 years (*SD*: 11.86), without a diagnosis of BPD.

### 2.2. Procedure

In this cross-sectional observational study, an online survey was administered to a group of individuals from the general population, as well as to another group who reported having received a diagnosis of BPD from a medical professional and were undergoing DBT. It was conducted in January 2021 during the COVID-19 pandemic. The survey administration was conducted online in a self-administered format. The general population sample was recruited through mass messages on social networks. BDP patient recruitment was intentional, at the “Terres de Lleida” Mental Health Centre (Spain), which specializes in DBT in individuals with BPD.

The study was conducted in accordance with the Declaration of Helsinki. This private center complies with the legal and ethical standards established by law. Its implementation did not deviate from the usual good clinical practices at the center for patients with BPD who agreed to participate. The Ethical Code of Psychologists, established by the General Council of Psychology of Spain, was applicable. Following recruitment, all participants were briefed on the study’s objectives and the confidentiality of their data. The survey posed no physical or mental risks to participants, who had the option to contact the principal investigator at any time. Participation was entirely voluntary and non-profit, with participants given the opportunity to withdraw from the study at any point. Prior to participation, individuals were required to provide informed consent. 

### 2.3. Applied Treatment

After being diagnosed, patients received DBT, an outpatient psychotherapy structured in weekly individual sessions and weekly group sessions for skills training. These were complemented with emergency phone coaching. Additionally, the therapist relied on weekly team consultations with other therapists to prevent burnout described in this type of therapy. The therapy was administered by the clinical psychologists of the mental health center. Both individual DBT therapists and DBT group therapists have experience in this type of therapy. They have all received prior training to apply DBT. Furthermore, during the therapy period, participants were periodically evaluated by experienced psychiatrists, ensuring adequate follow-up and comprehensive care.

A summary of the treatment protocol applied is shown in Table 1. The individual therapy, lasting one hour weekly, was initially focused on exploring parasuicidal behavior and emphasizing problem-solving behaviours and short-term distress tolerance techniques. Subsequent sessions addressed behaviours that interfere with therapy, such as agreement non-compliance, and possible negative or high-risk health-related behaviours. Additionally, reinforcing acquired behavioral skills was sought. Trauma history was addressed when the patient felt ready for it through verbalization and exposure techniques, constantly reinforcing the patient’s self-respect behaviours. Skills training was conducted in groups of 5 to 8 patients, in weekly sessions lasting two hours. Behavioral skills, unstable interpersonal relationships, impulsivity, emotional instability, and fear of COVID-19 following the onset of the pandemic were addressed. Patients received training in mindfulness techniques. After each session, tasks were assigned to patients to reinforce their skills. Diary cards were used to record the use of practiced skills for later analysis in individual sessions. The therapy was complemented with telephone counselling during crisis moments between sessions. It was applied as reinforcement for parasuicidal behaviours after reaching an agreement with patients that they should call the therapist before engaging in parasuicidal behavior. 

As recommended for this type of treatment, the therapist participated in weekly team consultations with other therapists, aiming to monitor treatment fidelity, enhance therapeutic skills, maintain motivation, and promote empathy and acceptance of the patients. Some of the challenges addressed during these sessions are common when applying this type of therapy. These include managing patient resistance, particularly with respect to compliance with treatment agreements and participation in therapy; challenges in balancing the timing and intensity of trauma-focused interventions to ensure patient safety; and how to implement the best intervention techniques in acute crisis situations between sessions. During the COVID-19 pandemic, supervisory discussions also focused on finding solutions to address fears and concerns related to the pandemic. As a result of these discussions, solutions were implemented, including refining therapeutic techniques and adjusting treatment plans to better fit individual patient needs. Another purpose was to guarantee continuous collaboration and communication within the therapeutic team, supporting each other, and improving self-care practices to prevent burnout. These supervision sessions play a crucial role in supporting the professional development of the therapist and maintaining the quality of care provided to patients throughout the treatment process.

### 2.4. Measures

The administered questionnaire included items aimed at gathering demographic data and scales such as the Fear of COVID-19 Scale (FCV-19S; [66]) to assess the contextual level of fear induced by the pandemic, the Goldberg Anxiety and Depression Scale (GADS; [67] to determine the levels of anxiety and depression within the sample, and the Positive and Negative Affect Schedule-Trait (PANAS; [68] to ascertain the levels of negative affect. These scales were utilized given the significant positive correlation established between fear and anxiety and depression [35,36,37,38,66], indicating a potential comorbidity pattern and suggesting cumulative levels of negative affect [69]. 

#### 2.4.1. Fear of COVID-19 Scale (FCV-19S; [66])

This scale assesses fear related to the coronavirus. It comprises 7 items rated on a five-point Likert scale (1 = strongly disagree; 5 = strongly agree). Example items include “I am unable to sleep because I’m worried about contracting COVID-19” and “I fear losing my life because of COVID-19”. Total scores range from 7 to 35, with higher scores indicating greater fear of the coronavirus disease. The scale reveals a Cronbach’s α of 0.84 for the total scale in the sample of this study. 

#### 2.4.2. Goldberg Anxiety and Depression Scale (GADS; [67])

This instrument is composed of two subscales of 9 binary (yes/no) items. The first subscale (1 to 9) is for anxiety, and the second subscale (19 to 18) for depression. Higher point values indicate a more severe problem, with 9 as the highest possible value for each subscale. In each scale, the first four questions are conditioning questions, because two affirmative answers are required to continue with the subscale, but in research the full scale is usually applied, and that is how it was used in the present study. The scale reveals a Cronbach’s α of 0.86 and 0.81 for the anxiety and depression subscales, respectively, in the sample of this study. 

#### 2.4.3. Positive and Negative Affect Schedule-Trait (PANAS; [68])

This questionnaire comprises 20 items divided into two subscales, measuring an individual’s positive and negative trait affect. The positive affect subscale includes items such as “I feel active and vigorous”, “I feel enthusiastic and full of energy”, and “I feel inspired and motivated”. The negative affect subscale includes items such as “I feel annoyed and angry”, “I feel scared and fearful”, and “I feel sad and discouraged”. It uses a 5-point Likert scale (1 = very slightly or not at all; 5 = extremely). Total scores are calculated by adding the scores of the subscale items. The scale reveals a Cronbach’s α of 0.85 and 0.90 for the positive affect and negative affect subscales, respectively, in the sample of this study. 

### 2.5. Statistical Analysis

The statistical analysis was conducted using the SPSS (version 27) statistical package. Firstly, demographic characteristics of the samples used in the study were examined through frequency analysis and descriptive calculations. In order to establish equivalence between the samples, their demographic characteristics were analyzed using Fisher’s test or ANOVA, as appropriate. Independent sample *t*-tests (for variables with two levels) were used to analyze age.

To assess the suitability of the variables used to measure the usefulness of DBT in the context of the study, Pearson bivariate correlations were derived, with the expectation of finding significant and positive correlations between them. 

Our hypotheses guided the subsequent statistical analysis. Hypothesis 1 posited that patients diagnosed with BPD, who have received fewer than 2 therapy sessions, would exhibit significantly higher levels of fear of COVID-19, negative affect, anxiety, and depression compared to both the healthy general population without a BPD diagnosis and patients with BPD undergoing long-term DBT (with more than 20 therapy sessions). Additionally, Hypothesis 2 anticipated that the BPD cohort undergoing long-term DBT would demonstrate levels of fear of COVID-19, negative affect, anxiety, and depression similar to those observed in the healthy general population without a BPD diagnosis. Consequently, the mean and standard deviation of each variable were determined as a measure of the level of fear of COVID-19, negative affect, anxiety, and depression. Subsequently, differences between groups for psychological measures were analyzed using ANOVA and Tukey post hoc analysis.

Taking into account the objectives and hypotheses of the study, regression analysis was not applied because it does not seek to explore the relationships between variables (e.g., the effect of fear of COVID-19 on other variables) or the incremental value of covariates (such as age and gender). This decision was, in turn, supported by the results obtained for the comparative analysis between groups reflected in the results section. 

## 3. Results

### 3.1. Sociodemographic Characteristics

The sociodemographic characteristics are presented in Table 2. As observed, there are no significant differences between groups for any of the variables, except for age. Differences of up to four years exist between the treated patient group and the general population group, with the least significant difference observed between treated and untreated patient groups (*t*-test: 0.73; *p* < 0.788). Despite this discrepancy, the groups can be considered comparable and suitable for the study. 

### 3.2. Psychological Variables

As can be seen in Table 3, as expected, the results obtained in the Pearson correlation analysis confirm the presence of significant and positive results between fear of COVID-19, negative affect, anxiety, and stress, validating the suitability of the variables used in this study.

The data obtained for the different measures are presented in Table 4. The analyses show that there are no significant differences between analyzed groups for COVID-19 fear (*F* = 0.686; *p* = 0.504) or for positive affect (*F* = 0.910; *p* = 0.404). However, significant differences are found for measures of depression (*F* = 17.525; *p* < 0.001), anxiety (*F* = 14.55; *p* < 0.001), and negative affect (*F* = 9.078; *p* < 0.001).

The post hoc analyses revealed the following:

a. For the measures of depression and anxiety, the differences were found between individuals from the general population without BPD and the two groups of BPD patients, among which there were no significant differences. Therefore, the treatment may not have succeeded in reducing the levels of anxiety and depression. 

b. For negative affect, higher scores were observed in the BPD group at the start of treatment. There were no significant differences between the long-term treated BPD group and the general population without BPD. Therefore, the treatment may have effectively lowered negative affect levels to those similar to those found in individuals without a diagnosis of BPD.

The results obtained after applying ANOVA indicate that there are no significant differences in terms of the demographic characteristics of gender, cohabitation, occupation, and education between the analyzed groups. The *t*-test indicates differences in the mean age of the groups, due to the older age of the healthy population group, but not between groups with PTL. There are also no significant differences related to the level of fear of COVID-19 between groups. Consequently, employing multiple linear regression analysis to examine the effect of fear of COVID-19 on the variables of negative affect, anxiety, or depression, or demographic covariates, was not considered necessary or relevant for this study.

## 4. Discussion

The findings confirmed the hypotheses posited in the study. The obtained results indicate that patients diagnosed with BPD who had undergone fewer than two therapy sessions exhibited significantly higher levels of negative affect compared to both the healthy general population, without a BPD diagnosis, and patients with BPD undergoing long-term DBT. Furthermore, the BPD cohort undergoing long-term DBT demonstrated levels of negative affect similar to those observed in the healthy group.

BPD has a high prevalence [1,2,3,4,5] and presents a series of significant challenges for both patients and the medical system, as extensively documented in the scientific literature [7,8]. Therefore, the results obtained in this study on BPD and its treatment with DBT in a real-life context capable of generating high emotional reactivity, such as the COVID-19 pandemic, are highly relevant.

The results show that the pandemic and its effects did not differentially affect the studied groups in terms of their fear of the virus or their ability to experience positive affect, but did have an impact on their mental health in terms of depression, anxiety, and negative affect. These findings are consistent with previous results, indicating that, during the COVID-19 pandemic, higher levels of depression and anxiety were recorded in psychiatric or mentally ill populations compared to the general population; this was associated with increased impulsivity and greater suicidal ideation [52,53,54], reflected in higher levels of negative affectivity in this case. The findings also agree with the results reported by other researchers [64,65] for patients with BPD, who recorded considerable mental distress, with negative feelings of anxiety, sadness, and helplessness, as well as symptoms of depression and negative thoughts.

No differences were observed in levels of fear of coronavirus between the study groups and the control group. The absence of significant differences in levels of fear of COVID-19 in patients with BPD undergoing short- and long-term treatment, compared to the general population, could be explained by the acceptance of restrictions and social isolation experienced by these patients, which may have provided feelings of safety from the virus [65]. On the other hand, the continued reinforcement of fear of the coronavirus due to ongoing exposure to pandemic-related information may have affected all groups equally in terms of fear reinforcement and its impact in terms of mental health. This explanation aligns with the results reported by Gu et al. [70] and Liu et al. [71], who observed that exposure to information about COVID-19 was a risk factor for anxiety and depression. 

However, it is important to note that levels of depression and anxiety did not show significant improvements in the long-term treatment group, suggesting limitations in DBT’s ability to fully address these symptoms in patients with BPD. This result contrasts with previous findings suggesting that DBT produces significant reductions in both state and trait anxiety [36,37,38]. In addition, other studies have advocated for the use of this therapy in patients with generalized anxiety, because of its ability to reduce suicidal ideation and address disorder-specific cognitive maintenance factors, including the perception of lack of control, negative social skills, increased self-focused attention, rumination, and avoidance [72]. This non-significant difference in anxiety and depression levels can be attributed to several factors. Firstly, the study was conducted during one of the waves of the pandemic, when in-person services were provided under strict health measures to prevent contagion, potentially leading to greater psychological discomfort. An inability to label emotions and increased psychological distress are characteristic features of BPD patients, particularly during periods of increased stress, such as the COVID-19 pandemic [60]. Individuals with BPD have a lower tolerance for distress [61], and studies indicate that the perceived risk of COVID-19 infection contributes to anxiety and distress intolerance in this population [62]. On the other hand, although social distancing measures implemented to prevent contagion may have fostered feelings of safety, they could also have increased feelings of loneliness, isolation, and depression [65]. Consequently, although feeling safe from the risk of contagion due to imposed social isolation, BPD patients may have experienced similar levels of fear of COVID-19 as the healthy population. However, concerns related to the pandemic and their lower tolerance for stress may have led to higher levels of anxiety and distress, exacerbating their pre-existing symptoms, and thus limiting the effectiveness of the treatment.

These findings underscore the need for effective interventions to address the mental health of individuals with BPD, particularly in highly stressful situations such as the pandemic. In this context, DBT has been identified as one of the most effective interventions for BPD, and this study supports its efficacy by finding that patients receiving long-term treatment showed levels of negative affect similar to those of the healthy general population, without a BPD diagnosis, at the end of treatment. However, it is important to note that depression and anxiety levels did not show significant improvements in the long-term treatment group. This suggests that there may be limitations in DBT’s ability to fully address these symptoms in BPD patients exposed to long-term stressful and traumatic situations, such as a pandemic. 

### 4.1. Limitations

This study has several limitations. Firstly, participants were recruited from a single mental health facility, potentially leading to a biased sample that may not accurately represent individuals with BPD in broader contexts. Consequently, the groups formed are similar in terms of gender, cohabitation, occupation, and education, but they are different in size, and the average age of the group of healthy people is somewhat older than that of the groups with BPD. Additionally, the three study groups (BPD patients receiving long-term treatment, BPD patients in the initial phase of treatment, and the healthy general population) exhibit similarity in the proportion of men and women; however, it is notable that the number of men is significantly lower compared to that of women, potentially constituting a limitation of the study. This gender disparity should be acknowledged in future research endeavors to ensure a more balanced representation. Furthermore, the study was conducted during a specific time period, which could restrict the generalizability of the findings to other populations or settings.

Secondly, the absence of regression analysis in the study could be considered a limitation, as this type of analysis would have allowed for a more comprehensive investigation of treatment-dependent differences in anxiety and depression, taking into account the influence of COVID-19 fear and other relevant variables. However, we chose not to conduct this analysis for several reasons. Firstly, we found that COVID-19 fear did not significantly vary among the established groups in the study, suggesting that this factor would not have a differential impact on anxiety and depression among the groups. Additionally, the study groups and variables were clearly defined and grounded in existing literature, obviating the need for further identification of factors through regression analysis. Furthermore, the methodologies employed, such as correlation analysis and ANOVA, were deemed suitable to meet the research objectives and test the hypotheses, providing sufficient understanding of the relationships between the variables of interest. In summary, although the inclusion of regression analysis would have been beneficial in terms of comprehensiveness, we considered it unnecessary and irrelevant given the specific characteristics of our study and the results obtained. In future studies, conducting regression analysis may be of interest to further explore the relationships between the variables of interest and gain a deeper understanding of the factors influencing anxiety and depression in the context of borderline personality disorder (BPD) amid a pandemic. Despite the lack of variation in COVID-19 fear among the established groups in our study, it is possible that this variable may have a differential impact in future studies, especially when considering other moderating or mediating factors.

Thirdly, the cross-sectional design employed in the study limits the ability to establish causality or examine the long-term effects of DBT on BPD symptoms. Furthermore, reliance on self-report measures introduces biases such as social desirability and recall bias. Fourthly, while the study assessed depression, anxiety, and negative affect, it did not investigate other relevant factors like quality of life, social functioning, or BPD-specific symptoms, which could offer a more comprehensive evaluation of treatment effectiveness. Lastly, the study lacks discussion on measures of treatment fidelity or compliance with the DBT protocol, as well as considerations of medication use, comorbid psychiatric conditions, or prior treatment history, which could potentially influence the observed results. 

### 4.2. Clinical Implications

These findings have significant clinical implications. Firstly, they underscore the importance of effectively identifying and treating BPD, tailored to the specific context. Secondly, they suggest that while DBT may effectively reduce negative affect in patients with BPD, additional interventions may be required to address depression and anxiety more comprehensively. 

### 4.3. Future Research Directions

Furthermore, these results emphasize the necessity of researching and developing specific interventions to address the mental health needs of individuals with BPD in situations of acute stress, such as a pandemic. Future investigations could aim to address the aforementioned limitations. Conducting further research employing longitudinal designs, objective assessments, and more diverse sampling strategies would enhance the generalizability of the findings and advance the understanding of the long-term impact of DBT on BPD.

## 5. Conclusions

Despite the identified limitations of the study, the results underscore the effectiveness of DBT in reducing negative affect among patients diagnosed with BPD. However, it is noteworthy that there may be constraints in DBT’s capacity to address concurrent depression and anxiety. These findings highlight the importance of accurately diagnosing and treating BPD, particularly during periods of heightened stress.

## Figures and Tables

**Table 1:** Summary of applied treatment protocol for borderline personality disorder (BPD) using dialectical behavior therapy (DBT).

Treatment Modality	Description	Examples of Techniques Used
Individual Therapy	Weekly one-hour sessions focused on exploring and addressing parasuicidal behaviours, compliance issues, and behavioral skills.	- Exploration of parasuicidal behaviours. - Teaching problem-solving skills. - Short-term distress tolerance techniques.
Skills Training	Weekly two-hour group sessions focusing on developing behavioral skills, stable relationships, impulsivity management, emotional stability, and coping with COVID-19 fears.	- Development of behavioral skills. - Teaching mindfulness techniques. - Impulsivity and emotional stability management. - Addressing COVID-19 fears.
Phone Coaching	Emergency phone consultations to regulate suicidal or maladaptive behaviours and facilitate skill generalization.	- Establishing clear rules for phone contact. - Teaching crisis resolution techniques. - Reinforcing skills during phone contact.
Team Supervision Meeting	Weekly meetings with other therapists to monitor treatment fidelity, enhance therapeutic skills, and maintain motivation and empathy towards patients.	- Clinical case analysis. - Peer support and supervision. - Prevention of burnout syndrome.

**Table 2:** Sociodemographic characteristics of the study sample.

	Group A (*n* = 69)	Group B (*n* = 30)	Group C (*n* = 188)	Differential Statistics
Age (M/*SD*)	37.33 (12.32)	39.63 (12.16)	41.15 (11.86)	*F* = 63683 *p* < 0.001
Sex				
Male	15 (21.7%)	6 (20%)	43 (22.9%)	*X*^2^ = 0.140 *p* = 0.93
Female	54 (78.3%)	24 (80%)	145 (77.1%)	
Coexistence				
Not living alone	59 (85.5%)	27 (90%)	171 (91%)	*X*^2^ = 2.806 *p* = 0.59
Living Alone	10 (14.5%)	3 (10%)	17 (9%)	
Occupation				
Currently working	54 (78.3%)	23 (76.7%)	161 (85.6%)	*X*^2^ = 3.834 *p* = 0.43
Out of work due to the pandemic	5 (7.2%)	3 (10%)	13 (6.9%)	
Out of work for other reasons	10 (14.5%)	4 (13.3%)	14 (7.4%)	
Education				
Basic education or no studies	1 (1.4%)	1 (3.3%)	4 (2.1%)	*X*^2^ = 0.942 *p* = 0.92
Vocational training	10 (14.5%)	3 (10%)	29 (15.4%)	
University education	58 (84.1%)	26 (86.7%)	155 (82.4%)	

Note: Group A: long-term therapy group (with more than 20 therapy sessions); Group B = patients with fewer than two therapy sessions; Group C: general population as healthy group. M = Mean; SD = Standard deviation.

**Table 3:** Bivariate correlations between the health indicators analyzed.

	Fear of COVID-19	Anxiety	Depression	Positive Affect	Negative Affect
Fear of COVID-19	1				
Anxiety	0.46	1			
Depression	0.34	0.69	1		
Positive affect	0.14	−0.01	−0.18	1	
Negative affect	0.56	0.62	0.53	0.15	1

Note: All correlations were significant at the 0.01 level (two-tailed).

**Table 4:** Psychological data and differential statistics.

	Group A (*n* = 69) Mean (*SD*)	Group B (*n* = 30) Mean (*SD*)	Group C (*n* = 188) Mean (*SD*)	Differential Statistics
Fear of COVID-19	11.11 (3.82)	11.78 (4.17)	10.85 (3.89)	*p* = 0.50
Anxiety	6.26 (2.63)	6.93 (2.40)	4.53 (3.03)	*p* < 0.001
Depression	4.54 (2.80)	4.93 (3.53)	2.77 (2.38)	*p* < 0.001
Positive affect	12.63 (5.56)	13.47 (7.80)	14.00 (7.16)	*p* = 0.40
Negative affect	12.61 (8.33)	16.70 (8.91)	10.24 (7.72)	*p* < 0.001

Note: Group A: long-term therapy group (with more than 20 therapy sessions); Group B = patients with less than 2 therapy sessions; Group C: general population as healthy group. M = Mean; SD = Standard deviation.

## Data Availability

The datasets analyzed in this manuscript are not publicly available due to ethical and legal restrictions. For further information regarding access to the data, please contact the corresponding author.
